# Carotenoids in Human Skin In Vivo: Antioxidant and Photo-Protectant Role against External and Internal Stressors

**DOI:** 10.3390/antiox11081451

**Published:** 2022-07-26

**Authors:** Maxim E. Darvin, Jürgen Lademann, Jörg von Hagen, Silke B. Lohan, Harald Kolmar, Martina C. Meinke, Sora Jung

**Affiliations:** 1 Center of Experimental and Applied Cutaneous Physiology, Department of Dermatology, Venerology and Allergology, Charité-Universitätsmedizin Berlin, Corporate Member of Freie Universität Berlin and Humboldt-Universität zu Berlin, Charitéplatz 1, 10117 Berlin, Germany; maxim.darvin@protonmail.com (M.E.D.); juergen.lademann@charite.de (J.L.); silke.lohan@charite.de (S.B.L.); sora.jung@charite.de (S.J.); 2 Institute for Organic Chemistry and Biochemistry, Technical University of Darmstadt, Alarich-Weiss-Straße 4, 64287 Darmstadt, Germany; kolmar@biochemie-tud.de; 3 Merck KGaA, Frankfurter Straße 250, 64293 Darmstadt, Germany; joerg.von.hagen@merckgroup.com

**Keywords:** beta-carotene, lycopene, zeaxanthin, stratum corneum, antioxidant status, free radicals, reactive oxygen species, lipid oxygen species, aging, nutrition, diet, fruit and vegetables, Raman spectroscopy, diffuse reflectance spectroscopy, palmoplantar erythrodysesthesia, hand-foot syndrome

## Abstract

The antioxidant system of the human body plays a crucial role in maintaining redox homeostasis and has an important protective function. Carotenoids have pronounced antioxidant properties in the neutralization of free radicals. In human skin, carotenoids have a high concentration in the stratum corneum (SC)—the horny outermost layer of the epidermis, where they accumulate within lipid lamellae. Resonance Raman spectroscopy and diffuse reflectance spectroscopy are optical methods that are used to non-invasively determine the carotenoid concentration in the human SC in vivo. It was shown by electron paramagnetic resonance spectroscopy that carotenoids support the entire antioxidant status of the human SC in vivo by neutralizing free radicals and thus, counteracting the development of oxidative stress. This review is devoted to assembling the kinetics of the carotenoids in the human SC in vivo using non-invasive optical and spectroscopic methods. Factors contributing to the changes of the carotenoid concentration in the human SC and their influence on the antioxidant status of the SC in vivo are summarized. The effect of chemotherapy on the carotenoid concentration of the SC in cancer patients is presented. A potential antioxidant-based pathomechanism of chemotherapy-induced hand-foot syndrome and a method to reduce its frequency and severity are discussed.

## 1. Redox Reactions, Free Radicals, and Antioxidants in the Human Skin

Redox reactions accompany the life cycle of all aerobic and anaerobic organisms and involve the redistribution of electrons between an oxidant atom (electron acceptor) and a reducing atom (electron donor) in the mitochondrial respiratory chain of the cells [1]. Free radicals can be particles, atoms, or molecules that have one or more unpaired electrons on the outer valence shell. This condition makes free radicals chemically very reactive, as they try to regain the missing electron by abstracting it from the surrounding molecules [2]. Most free radicals contain oxygen, so they are also called reactive oxygen species (ROS). The most common ROS in the biological tissues and organs are superoxide anion radicals (O_2_^•−^), hydroxyl radicals (^•^OH), hydrogen peroxide (H_2_O_2_), and singlet oxygen (^1^O_2_) [3]. But also reactive nitrogen species (RNS) [4] and lipid oxygen species (LOS) can be developed, such as lipoperoxynitrite radicals (LOO^•^), causing lipid peroxidation reaction cascades, inflammatory responses, and DNA damage [5,6]. Oxidized lipids can be toxic and act as lipid radicals or oxidants [7,8]. The interaction of hydrogen peroxide with iron (Fenton reaction) causes chronic inflammation [9]. The interaction of ROS and RNS with biological molecules and cells is capable of causing irreversible damage to their structure, which may lead to cellular dysfunction or even death. Most of the oxygen that is consumed by cellular mitochondria in the multistep processes of oxidative phosphorylation is converted into water, while a small fraction can diffuse out of the respiratory chain as ROS and RNS. ROS and RNS are able to permeate stratum corneum (SC) [10].

The formation of free radicals and oxidants is a physiological norm: free radicals contribute to a signaling function between cells and, together with oxidants, are used by cells to eliminate pathogens, such as viruses and bacteria [6,11]. Thus, free radicals play a dual role, as on the one hand they can cause oxidative damage and tissue dysfunction, but on the other hand they also serve as a natural defense against pathogens and as molecular signals activating stress responses that are beneficial for the organism. Mitochondria have been thought to both play a major role in tissue oxidative damage and dysfunction and provide protection against excessive tissue disorder through several mechanisms, including stimulating the opening of permeability transition pores [12]. However, the formation of ROS and RNS is not limited to mitochondria. It is modulated by various enzymatic or chemical reactions within various cell organelles such as lysosome, endoplasmic reticulum, peroxisome and cytoplasm. This makes a general treatment to prevent free radicals a very complicated task [13]. In addition, the imbalance between peroxisomal ROS/RNS production and removal may possibly damage biomolecules, perturb cellular thiol levels, and thus is a stress-triggered crosstalk between peroxisomes and mitochondria [14]. As a result, the oxidative and nitrosative stress in autophagy can be harmful to both cellular biomolecules and the signal mediator through reversible post-translational modifications of thiol-containing proteins [15]. Here, the balance is important to ensure the healthy crosstalk of the organelles. This assures proper cellular signaling and finally organ function [16]. At physiological conditions, the amount of ROS in the skin is higher than that of lipid radicals, and their total concentration does not exceed the threshold value of ~3.5 × 10^12^ radicals/mg. However, under pathological conditions or stress factors, the amount of lipid radicals becomes higher than the amount of ROS and their total concentration increases and exceeds the threshold value [8]. By combining a spin probe and a spin trap, Lohan et al. [17] recently demonstrated an increase in LOS concentration in skin that was irradiated with UVA light. Thereby, it was possible to distinguish between pre-stressed and unstressed skin by the ratio of ROS and LOS. A certain degree of stress is necessary to detect an inversion in the ratio of ROS and LOS, this reversal indicates an imbalance in the redox status.

To combat unwanted free radical leakage, cells, bio tissues, and organs contain different concentrations and mixtures of antioxidants—substances that effectively neutralize free radicals. Most antioxidants are able to neutralize several free radical attacks before being destroyed [18,19,20]. Some combinations of antioxidants, such as carotenoids and vitamin E [21], carotenoids and polyphenols [22], superoxide dismutase and catalase [23,24], lycopene, β-carotene, and vitamins C and E [25,26], act in synergy, significantly increasing the number of neutralized free radicals and thus improving the efficiency of antioxidant protection. The synergistic effect of antioxidants is a complex, multistep biochemical process, becoming manifest, for example, in plants, fruits, and vegetables, where the composition and concentration of antioxidants are optimally balanced to effectively neutralize free radicals [27,28]. Thus, the potential negative effect of free radicals and especially ROS on bio tissues and organs is minimized.

Normally, bio tissues and organs have a reserve of antioxidants so that redox homeostasis is maintained. However, this equilibrium can be disturbed by various stress factors, such as excessive solar radiation (mainly in the UV spectral range), X-rays, illness, medication, and lifestyle habits, such as smoking and alcohol consumption or a sleep deficit. Prolonged exposure to a stress factor increases the number of free radicals leading to the reduction of the concentration of antioxidants to a critical value, thereby promoting the development of oxidative stress. Oxidative stress leads to a change in the redox status of the cells, damage of cell compartments and, as a result, a disruption of homeostasis and damage of the affected bio tissue and organs [29,30]. The scientific community agrees that the accumulation of oxidative stress-induced damage may be the cause of premature aging [31,32,33] and underlies the development of a whole range of diseases, including cardiovascular and neurodegenerative diseases, diabetes, and cancer [34,35,36,37,38,39,40]. Thus, antioxidants protect biological objects against oxidation-induced injury and destruction.

**Figure 1:**
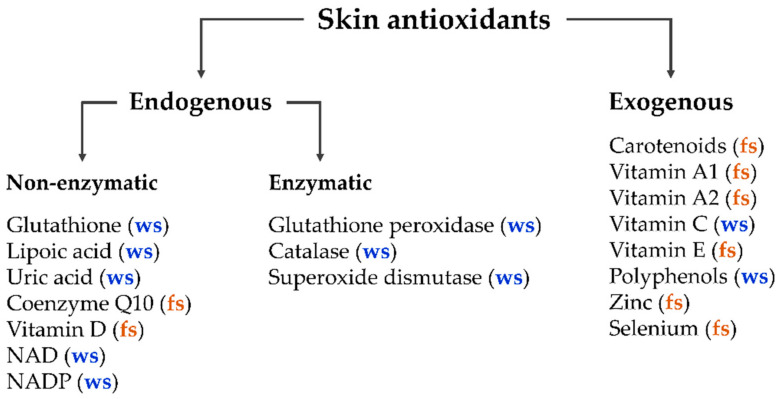
Classification of antioxidants in the skin. “**ws**”—water-soluble and “**fs**”—fat-soluble antioxidants.

The human body in general and the skin in particular contain a balanced set of antioxidants, which can be divided into two main classes—endogenous and exogenous antioxidants [23,41]. The major enzymatic endogenous antioxidants include glutathione peroxidase, catalase, and superoxide dismutase [24,42,43]. Non-enzymatic endogenous antioxidants include glutathione, lipoic acid, uric acid, coenzyme Q10, vitamin D, intracellular reducing agents nicotinamide adenine dinucleotide (NAD), and nicotinamide adenine dinucleotide phosphate (NADP). Exogenous antioxidants enter the human organism only by nutrition (dietary antioxidants), such as carotenoids; vitamins A1, A2, C, and E; polyphenols; zinc; and selenium [23,44,45,46,47,48]. Additionally, antioxidants can be divided into water-soluble and fat-soluble antioxidants, located in an “aqueous” or “lipid” environment, respectively (Figure 1).

The existing methods to measure antioxidants in human skin are divided into invasive, minimally invasive, and non-invasive methods. Invasive methods require a surgical intervention and the preparation of skin biopsies with subsequent chromatographic analysis of the skin samples [49,50]. Minimally invasive methods use a combination of methods for chemical and spectral analysis of the superficial SC that is removed by tape stripping or cyanoacrylate stripping techniques [51], as well as using potentiometric analysis of a gel/fluid that is extracted from the skin [45]. Invasive and minimally invasive methods to determine antioxidant concentrations are time-consuming and not accurate due to their inability to exclude contact of the biopsy material with oxygen and thus prevent the oxidation of antioxidants. The analysis of the skin samples is conducted ex vivo and not under physiological conditions. The advantage of non-invasive biopsy-free in vivo methods of measuring antioxidant content in the skin is obvious.

Human skin, as a metabolically active organ, contains a balanced set of enzymatic and non-enzymatic antioxidants. Their concentration may vary with the seasons [51,52] and also depends on lifestyle habits [43,52]. All of the known methods for determining the concentration of enzymes in the skin are invasive or minimally invasive [45,51]. The mechanisms of SC saturation with enzymatic antioxidants are not well understood; however, superoxide dismutase and catalase are known to be non-homogeneously distributed throughout the human SC, having a characteristic minimum near the surface [42,51]. Non-enzymatic endogenous intracellular reducing agents are mainly present in the viable epidermis and in the dermis in various concentrations. The SC contains significantly lower concentrations of enzymatic and non-enzymatic endogenous antioxidants. Thus, non-enzymatic exogenous antioxidants predominate in the SC, continuously entering the cells of the viable epidermis from the bloodstream and saturating the entire SC in the course of keratinization. However, this pathway is not unique, as vitamin E [53,54], the vitamins A1 and A2 [47], and carotenoids [55,56] reach the skin surface as part of the sweat and/or sebum secretion and thus increase antioxidant concentrations in the superficial SC [55,57,58]. The synergistic effect of all SC antioxidants determines the antioxidant status of the SC.

## 2. Carotenoids in Human Skin

Carotenoids are fat-soluble non-polar poly-unsaturated compounds of the terpene series, containing a polyene chain with cyclohexane rings or aliphatic isoprenoid residues at the ends with a highly delocalized π-electron system [59] and can be further categorized into two classes—xanthophylls (more polar) containing oxygen and carotenes (non-polar hydrocarbons), which do not contain oxygen [60]. Most carotenoids contain 40 carbon atoms and are capable of neutralizing ROS due to their antioxidant properties as shown in vitro in numerous studies [11,61,62,63,64,65,66,67]. The proximity of energies of triplet state of carotenoids and singlet oxygen state determines the antioxidant properties of carotenoids, which are related to the preferential chemical interaction of ROS with molecules having more than 10 π-π-conjugated C=C double bonds (alternation of double C=C and single C-C bonds) [68], of which there are 10 in lutein and 11 in carotenes, lycopene, and zeaxanthin [62]. Thus, the antioxidant efficacy of carotenoids is determined by the number of π-π-conjugated C=C double bonds [64,69,70,71]. In carotenes two π-π-conjugated C=C double bonds are located in the ionone rings, which reduce the effective conjugation length to 9.2–9.6 C=C bonds [72,73], thus weakening the rate constant in ROS neutralization by carotenes compared to lycopene [20,74]. Among carotenoids, lycopene has the highest antioxidant efficiency [74,75], followed by α-carotene, β-cryptoxanthin, β-carotene, zeaxanthin, and lutein [76]. Furthermore, carotenoids are natural filters of high-energy visible light, because due to the presence of more than 10 π-π-conjugated C=C double bonds they effectively absorb light in the violet-green spectral range [62,77].

Biosynthesis of carotenoids is carried out in visible light in the presence of oxygen by some bacteria, fungi (yeast), algae, corals, and higher photosynthetic organisms. The human organism does not synthesize carotenoids [78,79]; therefore, the only way to obtain them is through a diet that is rich in carotenoids, such as fruit and vegetables [80,81,82]. In the human body, apart from the skin, carotenoids are found in the blood plasma and are constantly circulating in the bloodstream [83,84]; they are also stored in the subcutaneous fatty tissue [47,85] and the liver [86]. The skin carotenoids α-carotene, β-carotene, and γ-carotene are pro-vitamin-active—their oxidation can result in the formation of vitamin A1 (retinol). The β-carotene molecule shows a unique feature, since two vitamin A1 molecules can be formed by splitting it [87,88]. The pro-vitamin properties of β-carotene and its oxidative conversion to vitamin A1 are common in humans and animals [89,90].

The predominant forms of vitamin A are retinol (vitamin A1) and dehydroretinol (vitamin A2) [47], which can be metabolized in the skin to retinal and further to retinoic acid [91], which has a broad therapeutic spectrum in dermatology, i.e., against acne and other inflammatory diseases. In addition to its keratolytic and anti-inflammatory effects, it exhibits antioxidant properties [46,92]. Retinol can exhibit pro-oxidant properties as it is easily oxidized and can form toxic products. Vitamin E prevents the oxidation of retinol. Vitamin A is not synthesized by the human body and enters the skin either by nutrition, by medication, after oxidation of pro-vitamin-active carotenoids, or after topical application of cosmetic or medical products [93,94].

In the human skin, the highest concentration of carotenoids is found within adipocytes in the fat-rich subcutaneous tissue [95,96] and in the SC within the lipid lamellae [55,97]. The carotenoid concentration is non-homogeneously distributed in the SC and has two maxima—near the surface and near the bottom of the SC [55,98], which is explained by the two independent delivery pathways: from the inside due to keratinization and blood circulation and from the outside with sweat and/or sebum secretion [56,57,58,99].

Analysis of human skin biopsies using high-pressure liquid chromatography reveals the presence of the following carotenoids: α-carotene (8 ng/g), β-carotene (26 ng/g), γ-carotene (7 ng/g), ξ-carotene (13 ng/g), lutein/zeaxanthin (9 ng/g), lycopene (69 ng/g), and phytoene/phytofluene (79 ng/g) [49,100]. Cutaneous carotenoids phytoene and phytofluene (3 and 5 π-π-conjugated C=C double bonds, respectively) absorb the light exclusively in the UV spectral range [62,70] and could not be detected in the SC in vivo by optical non-invasive methods. Lycopene, carotenes, and lutein/zeaxanthin account for approximately 60% of all SC carotenoids (the remaining approximately 40% are phytoene and phytofluene) [49]. Due to the presence of more than 10 π-π-conjugated C=C double bonds, they absorb light in the blue spectral range [70] and, therefore, only these carotenoids can be determined in the SC in vivo using non-invasive optical methods. The concentration of carotenes and lycopene exceeds 90% of all carotenoids that are detectable in the human SC by optical methods [49]. Β-carotene has the highest concentration among all carotenes, therefore, it is often noted in the literature that the major SC carotenoids are β-carotene and lycopene (both together over 70% of all skin carotenoids absorbing light in the violet-green spectral range).

## 3. Methods of Non-Invasive In Vivo Determination of Carotenoids in the SC

Carotenoids can be determined by analyzing skin biopsies using high-pressure liquid chromatography [49,85], but this method is highly invasive and does not provide accurate information due to preparation-induced oxidation of carotenoids. Non-invasive methods to assess the carotenoid content in the SC in vivo are advantageous and currently popular in skin research due to their practicability and reliability. The most effective non-invasive methods to measure carotenoids in the human SC are limited to optical methods, which include resonance Raman spectroscopy (RRS), confocal Raman micro-spectroscopy (CRM), skin color measurements, and diffuse reflectance spectroscopy, reviewed in detail in [101].

As the concentration of carotenoids in the SC is the highest compared to viable epidermis and dermis skin layers [47], it is advantageous to measure on the palms of hands, which provide a thick SC and are easily accessible [102]. Skin on other body areas, such as the forearm and face, are also suitable for in vivo measurements using exemplary methods.

### 3.1. Resonance Raman Spectroscopy (RRS)

The non-invasive in vivo measurement of carotenoids in human skin was first demonstrated by Hata et al. [49] using resonance Raman spectroscopy (RRS). The Raman band intensity at ≈1524 cm^−1^ corresponding to the C=C vibration of carotenoids was analyzed under the excitation at 488 nm. A two-wave excitation scheme was realized for the separate determination of carotenes and lycopene [100,103] and adapted for the measurement of lycopene in the SC [104]. Furthermore, an RRS-based prototype device was developed and tested for non-invasive in vivo measurements of carotenoids in the human SC, which was characterized by the shorter measurement time of ≤5 s, and a stable RRS signal that was achieved by using an excitation laser spot extended to approximately 6.5 mm on the skin, allowing to reduce the influence of skin heterogeneity and pigmentation [77,104]. This RRS device was used in many in vivo studies that were mostly applied on the palms of the hands and the forearms.

Practically, in some cases, the fluorescence background that is always presented in Raman spectra is too high to detect the Raman band at ≈1524 cm^−^^1^. In this case, the effect of fluorescence photobleaching is used: The skin is irradiated with probing radiation continuously for 1–3 min to reduce the skin fluorescence intensity [105]. Fluorescence photobleaching is evident in all the skin layers and for the broad range of excitation wavelengths [106] and has no effect on the position and intensity of the Raman bands [105]. An alternative technique is the complete subtraction of the fluorescence background using shifted excitation resonance Raman difference spectroscopy (SERRDS), which is based on the sequential excitation of the skin at two closely located laser excitation wavelengths (487.2 and 487.6 nm) and the subsequent subtraction of the obtained spectra and restoration of the Raman band [107]. The main disadvantages are increased device costs and a longer measurement time.

### 3.2. Confocal Raman Micro-Spectroscopy (CRM)

For the non-invasive in vivo determination of natural and topically applied carotenoids, a depth profile in the SC can be obtained using a confocal Raman microscope (RiverD International B.V. model 3510 SCA, Rotterdam, The Netherlands) [57]. Measurements can be taken on any skin site that is suitable for positioning on the CRM with high spectral and spatial resolution (2 cm^−1^ and ≤5 µm, respectively) to determine the depth profiles of the components and physiological parameters of the SC in vivo [108,109,110]. The Raman spectrum of the SC in the 400–2000 cm^−1^ region is excited at 785 nm and contains the intense Raman bands of the main SC components, such as proteins, lipids, natural moisturizing factor molecules, and carotenoids. The excitation wavelength at 785 nm is not resonant for excitation of the carotenoids but provides pre-resonance excitation. The carotenoid-related Raman band at ≈1524 cm^−1^ does not overlap with other bands and can, therefore, be well detected and analyzed using different algorithms [55,57,98]. The skin fluorescence is minimal at an excitation of 785 nm and is mainly due to the melanin [111] and oxidation products of the SC components [112,113]. A detailed description of CRM has been provided previously [101,108].

### 3.3. Diffuse Reflectance Spectroscopy

The main disadvantages of the RRS and CRM methods to measure carotenoids in the SC are high costs of the devices and their immobility due to the bulky size and high sensitivity to vibrations. Therefore, in order to conduct in vivo measurements of the carotenoid concentration in the human SC outside the laboratory, a device that is small in size and resistant to vibrations was developed. It has the size of a computer mouse and was produced by the company Biozoom (Kassel, Germany) [114,115,116]. The measurements are based on the diffuse light reflectance spectroscopy method [117]. There are two technical implementations: using a stable mini-spectrometer [114] and using the multiple spatially resolved reflectance spectroscopy (MSRRS) method [116]. The MSRRS method has shown better measurement stability of carotenoids in the SC of the human palm and is used in the latest models of Biozoom devices. Due to the influence of the melanin and blood chromophores hemoglobin and bilirubin on reflection spectra, the measurement of carotenoids using the MSRRS method is limited to the human palm [116], where the SC thickness is >100 µm [102]. To minimize the influence of blood, the pressure-mediated reflectance spectroscopy method was introduced [118].

### 3.4. Skin Color Measurements

The color of human skin is mainly determined by melanin, carotenoids, and the blood chromophores hemoglobin and oxyhemoglobin [119]. Measurements of the yellow component of the color palette of the skin‘s diffuse reflection spectrum provides information on the concentration of carotenoids in the epidermis [120]. This method is characterized by the lowest stability and reproducibility in comparison with other methods as it is impossible to eliminate the influence of melanin and blood chromophores on the resulting skin reflection spectrum [101]. However, this method is frequently used by various scientific groups to determine the concentration of carotenoids in human skin in vivo [80,121,122,123,124], which is presumably due to the low costs of the device and its simplicity regarding measurements and result analysis.

## 4. Carotenoids Are Marker Substances of the Antioxidant Status of the Human SC In Vivo

**Figure 2:**
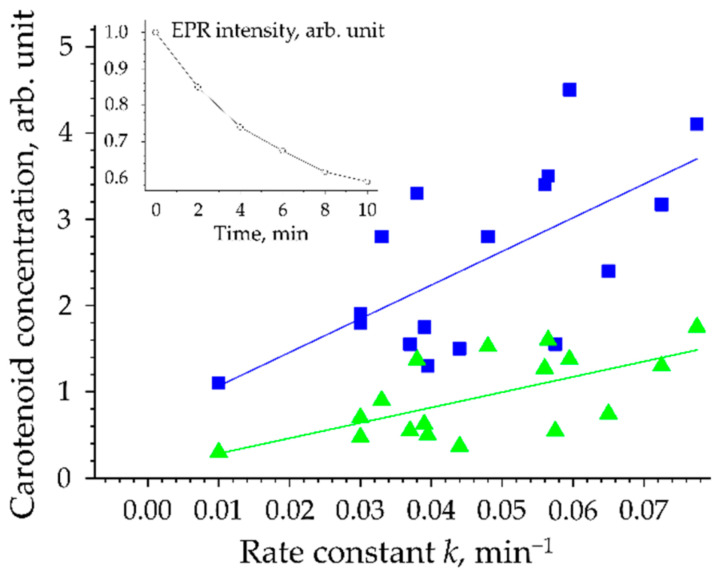
Dependence of all carotenoid concentrations (squares, blue line) and only lycopene (triangles, green line) on the rate constant *k* of radical marker TEMPO, measured in vivo in the inner forearm’s SC of 17 volunteers. The inset shows the dependence of natural attenuation of the EPR signal intensity of TEMPO radical marker in the human SC. The figure is adapted from [131].

The diversity of antioxidants in bio tissues and skin makes the determination of the concentration of each antioxidant separately a rather laborious and difficult task. For this reason, methods have been developed to determine the antioxidant status of bio tissues (primarily bio liquids) by considering the simultaneous action of all constituent antioxidants in the bio tissue/bio liquid as a complex response to the stress factor [125,126,127]. For example, the antioxidant status of blood can be determined by the concentration of protein and lipid peroxidation products as well as the concentration of constituent antioxidants such as vitamins A, C, E, and the carotenoids β-carotene and lycopene [46]. As noted previously, the human SC contains various antioxidants, acting synergistically, forming the antioxidant status of the SC. Determining the antioxidant status of the SC in vivo requires determining the efficiency of neutralizing free radicals. Electron paramagnetic resonance (EPR) spectroscopy is a non-invasive method for the detection of radical formation in tissues, organs, and cell cultures. The topical application of the spin probe nitroxide TEMPO (2,2,6,6-tetramethylpiperidine-1-yl-oxyl) on the skin allows statements about the redox state in the SC as shown for in vivo measurements on the skin of the human inner forearm [128]. Nitroxides are paramagnetic species that have a free, unpaired electron in the outer shell. This property leads to the fact that they provide a characteristic EPR signal by themselves, i.e., they are EPR-active. After reacting with a radical, they become EPR inactive, i.e., the intensity of the EPR signal decreases with increasing radical production. Thus, the intensity of the EPR signal is proportional to the concentration of the spin probe TEMPO in the SC and decreases exponentially with time (inset in Figure 2). A decrease in the spin probe concentration, according to Hee et al. [129,130], can be described by the following formula:

I (t) = I_0_ exp (–*k*t) (1)

where *k* is the radical marker quenching rate constant, which directly depends on the antioxidant status (total action of all antioxidants) of the SC, measured in min^−1^.

Thus, the higher the antioxidant status of the SC (higher antioxidant protection), the faster the neutralization of TEMPO and the faster the EPR signal intensity decreases and, consequently, the higher is the rate constant *k*. Conversely, the lower the antioxidant status of the SC is, the longer the lifetime of the spin probe and the lower is the rate constant *k*. Figure 2 shows the relationship between the rate constant *k* characterizing the antioxidant status of the SC and the concentration of all carotenoids (blue line) and only lycopene (green line) in the SC of 17 volunteers, measured in vivo by EPR and RRS.

A positive correlation (R = 0.65) between the concentration of carotenoids and the decrease of the skin probe TEMPO is given implying that the more carotenoids are in the SC, the higher the rate constant *k*, i.e., the higher is the antioxidant status of the SC. These dependencies confirm that carotenoids can be considered as marker substances of the entire antioxidant status of the human SC in vivo [131], which was indirectly confirmed in other studies [132,133,134]. However, recent investigations have shown that smokers may have a lower carotenoid concentration but higher *k* values in the SC. This was explained by the substantial increase of glutathione concentration in the upper SC of smokers compared to non-smokers and primarily the interaction of the skin probe TEMPO with the endogenous antioxidants [135]. Thus, in smokers, the skin probe TEMPO was reduced primarily by glutathione, resulting in a higher *k* value. The obtained results (Figure 2) are important for an easier investigation of the changes in the entire antioxidant status of the human SC in vivo based on the determination of the carotenoid concentration, which substantially extends the number of practical applications as further discussed in paragraph 5 and 6 below.

## 5. Factors Resulting in the Reduction of Carotenoids in the SC

External and internal stress-factors on human skin can result in the formation of free radicals which cause the decrease of antioxidant concentrations in all skin layers [132].

One of the main external stress factors is solar radiation, whose spectrum reaching the earth′s surface is in the 290–3000 nm range [136]. The effect of sunlight on the skin depends on the radiation dose and the penetration depth, which is minimal for UV and maximal for the IR-A spectral range. External mechanical irritation including microlesions that are caused by shaving seem to have no significant effect on the concentration of carotenoids in the human SC [137]. Air pollution by various gases and particulate matter has a negative effect on human skin [138] and can also act as an external stressor, leading to the formation of free radicals in human skin [139], whose effect is enhanced in the presence of UV radiation [140]. To protect the skin from the negative effects of external pollutants, topical application of antioxidants is effective [141]. Internal stressors include lifestyle habits such as smoking, alcohol abuse, high-intensity exercise, stress, and dietary habits, as well as diseases. The results of the effect of various stressors on carotenoid concentrations in the human SC in vivo have a strong practical interest to better understand the skin physiology, are presented below.

### 5.1. Effect of UV Radiation

**Figure 3:**
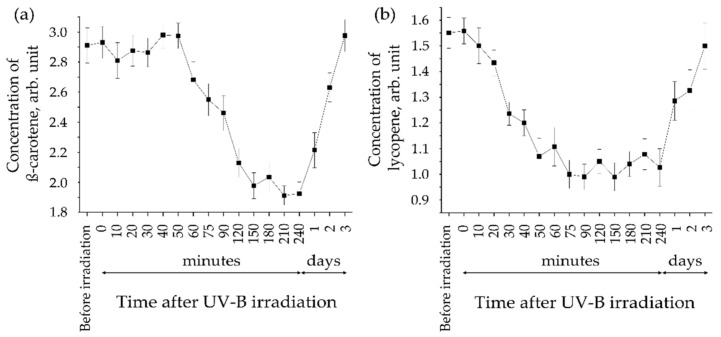
Changes in the average concentration of ß-carotene (**a**) and lycopene (**b**) in the SC of human inner forearm skin after UV-B irradiation (dose 0.03 J/cm^2^: light intensity 0.3 mW/cm^2^, irradiation time 100 s) measured in vivo by RRS. The figure is adapted from [147].

The negative effects of UV radiation (290–400 nm) on human skin have long been known [142] and can be divided into direct and indirect effects. By absorbing residues of the aromatic amino acids tryptophan, tyrosine, and phenylalanine, UV-B radiation (290–320 nm) has mainly direct effects, causing dimerization of DNA’s thymine, photoisomerization of urocanic acid and the development of erythema [143,144,145]. UV-A radiation (320–400 nm, approximately 6.3% of the total solar energy) causes mainly indirect effects, consisting of the formation of a large number of free radicals, primarily ROS, determined ex vivo on skin biopsies using EPR spectroscopy [3,146]. Tissue-tolerable plasma-induced UV radiation can be used for skin disinfection, which is accompanied by a reduction of the carotenoid concentration in the superficial SC [99]. Under the irradiation with UV-B (dose 0.03 J/cm^2^: light intensity 0.3 mW/cm^2^, irradiation time 100 s), a decrease of the carotenoid concentration in the human SC over time was demonstrated in vivo using RRS [147], as shown in Figure 3.

A decrease of the lycopene/ß-carotene concentration is observed 0–30/30–90 min after termination of irradiation (Figure 3), which is probably caused by different quenching rates in the neutralization of ROS. The maximal decrease in the carotenoid concentration was observed 2–3 h post-irradiation, which seems to be related to the induction of inflammatory reactions and the subsequent oxidative stress. The recovery of carotenoids in the SC to the initial level lasted up to three days [147]. EPR spectroscopy showed that the amount of ROS that formed in the human SC subsequent to UV exposure is considerably higher by approximately 3.5 times in the skin in vivo than in skin biopsies ex vivo, which can be explained by the higher oxygen saturation of viable skin cells by blood circulation compared to skin biopsies [148].

Sunscreens are often used to protect the skin from solar UV radiation [149]. They contain absorbers, reflectors, and scatterers of UV light, among which titanium dioxide and zinc oxide are the best known [150,151]. Plant extracts [152] and other antioxidants [153] are also used to enhance skin protection [154,155] and to increase the photo-stability of sunscreens [156].

### 5.2. Effect of Visible Light

**Figure 4:**
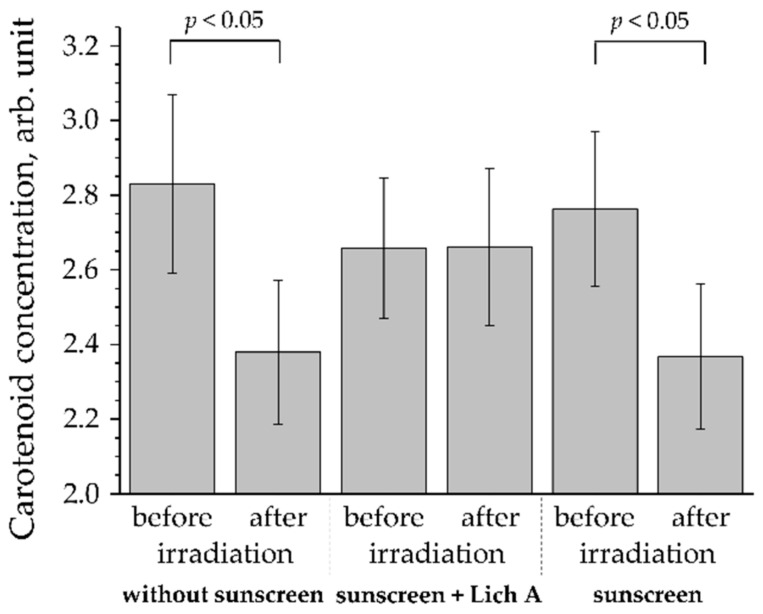
Changes in carotenoid concentration in the SC of the inner forearm skin of 10 volunteers after exposure to the violet-blue light (dose 100 J/cm^2^: light 40 mW/cm^2^, exposure time 42 min), measured in vivo by RRS. There are three scenarios that are shown: skin without the application of sunscreen, skin with the application of sunscreen with a sun protection factor 50 containing the antioxidant licochalcone A in a volume concentration of 0.005%, and skin with the application of sunscreen with sun protection factor 50 containing no antioxidants. The figure is adapted from [162].

Using EPR spectroscopy, Zastrow et al. [3] have demonstrated that the exposure of excised human skin to visible light (400–760 nm) results in the formation of free radicals. Later, using in vivo RRS, Vandersee et al. [157] showed that the concentration of carotenoids in the human SC decreased by 13–21% after irradiation with light of the violet-blue spectral range (400–495 nm, maximum intensity around 440 nm; dose 50 or 100 J/cm^2^: light intensity 100 mW/cm^2^, irradiation time 500 or 1000 s) (Figure 4).

A decrease in the carotenoid concentration was observed immediately after irradiation, and recovery to the initial value lasted between 2 up to 24 h, depending on the irradiation dose (50 or 100 J/cm^2^). It was suggested that violet-blue light, also known as high-energy visible light, causes ROS formation in the skin, which induces a decrease of the carotenoid concentration in the SC. Subsequently, the ROS formation in the skin that is caused by exposure to visible light was confirmed in an in vivo study of human skin using EPR spectroscopy [158] and in numerous studies evaluating oxidative stress-induced changes in the skin subsequent to irradiation with visible light [159,160,161].

To protect the skin from the effects of radiation in the visible spectral range, the use of standard sunscreens that protect in the UV spectral range is ineffective. The incorporation of filters into sunscreens that absorb, reflect, or scatter light in the visible spectral range is rarely used in practice because visible light filters frequently have a dense texture and are not transparent, thus adding an undesirable color to the cosmetic product and, after application, to the skin [163]. The addition of antioxidants increases the protective effect of sunscreens [164]. Recent human skin studies in vivo demonstrated that topical application of sunscreens (sun protection factor 50) containing *Glycyrrhiza inflata* root extract, namely the phenolic compound licochalcone A in a volume concentration of 0.005%, has a protective antioxidant effect on human skin (Figure 4) by neutralizing the violet-blue radiation-induced ROS [162]. As can be seen in Figure 4, the use of a sunscreen that has a high sun protection factor of 50, but contains no antioxidants, has no protective effect against violet-blue radiation on human skin. Thus, the addition of antioxidants to cream formulations is effective in neutralizing ROS, which was also confirmed previously [154,160].

### 5.3. Effect of Near-Infrared Radiation

In contrast to the comprehensively studied effects of UV radiation, the effects of near-IR radiation (IR-A 760–1440 nm and IR-B: 1440–3000 nm) on human skin have received less attention. Previously, it was thought that due to the low quanta energy (<1.6 eV), IR radiation would be unable to cause serious biochemical changes at the cellular level, and that its effect would be limited to heating the skin, which is caused by the strong absorption of IR radiation by the cutaneous water. Kligman [165] was the first to note that irradiation of the skin with IR and UV radiation causes similar morphological changes in the dermis, which was subsequently confirmed by other researchers [166,167]. Studies on cell cultures have shown that IR radiation is able to activate cell mitochondria and that the main chromophore-absorbing IR radiation is cytochrome c-oxidase, an enzyme in the mitochondrial respiratory electron transport chain forming ROS [168,169]. It is important to note that under physiological conditions cytochrome c-oxidase is bound to nitric oxide, which is an inhibitor of this enzyme. Under IR irradiation of the cell, the nitric oxide is released from the catalytic center of cytochrome c-oxidase, which leads to activation of the enzyme, enhancement of processes in the electron transfer chain, and the formation of ROS [170]. Schieke et al. [166,171] showed that IR radiation activates signaling pathways, in particular, mitogen-activated protein kinases and causes the expression of collagenase matrix metalloproteinases that are capable of degrading extracellular matrix proteins, which is also an indirect evidence of ROS formation due to IR irradiation. Another study of skin biopsies has shown that IR radiation is able to activate mitochondria of epidermal and dermal cells, causing ROS formation [172], which may cause temperature-independent biochemical changes at the cellular level. In vivo CRM reveals oxidation-like changes in the SC, viable epidermis and dermis after IR exposure [173].

**Figure 5:**
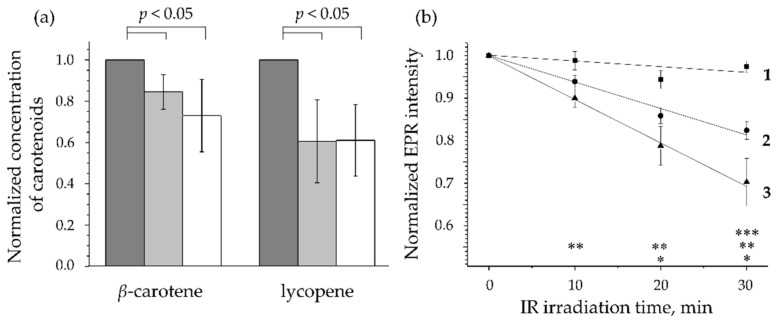
(**a**) Changes in the average normalized concentration of all carotenoids and lycopene in the SC of the inner forearm in 12 volunteers after irradiation with IR-A and IR-B light (white column) and IR-A light (gray column) in comparison with non-irradiated skin (black column), measured in vivo by RRS using an irradiation dose of 306–342 J/cm^2^: light intensity 170–190 mW/cm^2^ and an irradiation time of 30 min. (**b**) Dependence of the PCA radical marker EPR with shown intensity changes in the SC of non-irradiated porcine ear skin (curve 1), under irradiation of the skin with IR-A light (curve 2), and with IR-A and IR-B light (curve 3). An irradiation dose of 189–207 J/cm^2^, a light intensity of 105–115 mW/cm^2^, and an irradiation time of 30 min was applied. “*”, “**”, “***” are statistically significant differences for curves “1” and “2”, “1” and “3”, “2” and “3” (Wilcoxon test, *p* < 0.05). The figure is adapted from [177].

The decrease of the carotenoid concentration in the human SC after irradiation with near-IR light (600–3000 nm, intensity maximum around 1000 nm; dose 306–342 J/cm^2^: light intensity 170–190 mW/cm^2^, irradiation time 30 min) was shown in vivo using RRS [174] (Figure 5a). It was hypothesized that the decrease in the carotenoid concentration might be due to the neutralization of the formed ROS, and this assumption was later confirmed by a temperature-controlled ex vivo study on porcine skin using EPR spectroscopy [175]. These results were preceded by the work of Zastrow et al. [3], in which the free radical action spectrum of excised human skin was demonstrated—about 50% of free radicals (predominantly ROS) are formed in the skin by irradiation in the visible and near-IR spectral ranges. Figure 5b shows that ROS are formed in the skin as a result of irradiation in the IR-A and IR-B spectral ranges, which is confirmed by the immediate decrease of carotenoid concentration in the SC after the termination of irradiation [176]. Recovery to the initial value lasted up to 24 h and starts in the superficial SC depth, where carotenoids are delivered with sweat and/or sebum, where they are spread over the skin surface and can repenetrate into the SC [58].

To protect the skin from IR-A and IR-B radiation, the use of absorbers, reflectors, and scatterers as part of sunscreens is less effective compared to protection in the UV spectral range. An effective protection includes the topical application of antioxidants that neutralize ROS that has formed as a result of IR exposure [164,167,178]. Adding antioxidants to a cosmetic formulation also reduces the oxidation of its components [25] and, consequently, increases the storage time of the cosmetic products.

### 5.4. Effect of Solar Radiation

The results that are presented above show that irradiation of human skin with light in the UV, visible, and IR spectral range leads to a decrease of the carotenoid concentration in the SC, which is an indicator of ROS formation. The maximum ROS concentration in the skin is formed in the UV-A range and the minimum in the IR range [3,158]. However, the effect of solar radiation within a wide wavelength range 290–3000 nm on the skin may differ due to possible overlapping effects that are induced by different spectral regions [179].

The effects of solar radiation (2 h of daily irradiation for four weeks in the summer season on the inner forearm of volunteers permanently residing in Berlin (*n* = 12) and Monaco (*n* = 7)) on the concentration of carotenoids in the SC and collagen Type I in the papillary dermis were investigated in vivo non-invasively using RRS [77] and two-photon tomography [149], respectively. Before irradiation, the skin of the inner forearm of one arm was left untreated and the other forearm was treated with sunscreen (sun protection factor 30) containing antioxidants and protecting in a broad spectral range from UV-B to IR-B [149].

Sunlight exposure of the untreated skin was shown to cause an approximately 14% decrease in carotenoid concentration and an approximately 18% decrease in collagen Type I as average for all the volunteers (*p* < 0.05). In contrast, no significant change in the carotenoid concentration was observed in skin that was treated with sunscreen. These results demonstrate the photo-protective effect of sunscreen containing antioxidants on the epidermis and on the dermis in vivo.

The initial concentrations of carotenoids and collagen Type I in the skin of volunteers permanently residing in Monaco were significantly lower than in the skin of the permanent participants in Berlin (*p* < 0.05), which is explained by the significantly more intense exposure of the Monegasque participants to sunlight before starting the study [149].

### 5.5. Impact of Internal Stress Factors and Diseases

In addition to external, the action of internal stress factors on the human body causes the formation of free radicals, in particular ROS, which reduce the antioxidant capacity. Such stressors include human lifestyle, in particular smoking [180,181], alcohol abuse [182], excessive physical activity [183], and household/work stress [184,185]. ROS that are generated in the skin are neutralized by its own antioxidant system. In the case of prolonged exposure to a stressor, the concentration of ROS increases significantly, which leads to a decrease in the antioxidant concentration in the body and in the skin.

Using non-invasive RRS and diffuse reflectance spectroscopy it was shown in human skin in vivo that the consumption of high doses of alcohol [186], excessive physical activity [187], domestic and work stress [52,188,189], and other stress factors [189,190] result in a reduction of the carotenoid concentration in the human SC, which depends on the initial concentration of the carotenoids’ and stressor’s intensity. It has been noticed that the concentration of carotenoids in the SC of smokers is significantly lower than in non-smokers (*p* < 0.001) [135,191,192], which is in agreement with the literature data [50,193,194]. The concentration of carotenoids in the SC of women is significantly higher than in men (*p* < 0.05) [192]. At the same time, the concentration of carotenoids in the SC does not correlate with age but tends to decrease with increasing body mass index to values above 30, which is also consistent with published data [193,194,195]. In these cases, chronic diseases or psychological stress due to social exclusion might act as negative stressors.

It is known that various diseases can cause a disturbance of the redox balance, which leads to the formation of an enormous amount of free radicals and the development of oxidative stress that is accompanied by a decrease in the antioxidant concentration [5,6,196]. For example, cardiovascular [68,197], neurodegenerative [198], kidney [199], eye [200], lung [201], skin [202,203], including melanoma [204], and other diseases [205], including different kinds of cancer [206,207,208], are associated with the development of oxidative stress and affect the antioxidant status of the body.

A number of studies have shown an association between a diet that is rich in carotenoids, e.g., fruit and vegetables, and the risk of developing certain types of cancer according to the following principle: the more carotenoids in a diet, the lower the probability of cancer development [209,210,211]. The same relationship has been observed between a carotenoid-rich diet and the risk of cardiovascular [212,213], eye [214], and other diseases [11,215]. The inverse relationship for the development of lung cancer has been reported for heavy smoking individuals after supplementation of a high single dose of ß-carotene, which strongly exceeds the physiological concentration [68]. It should be taken into consideration that there are different antioxidant mechanisms of carotenoids including direct (energy transfer) and indirect pathways (via transcription factors Nrf2 and NF-κB and the activation of nuclear hormone receptor pathways RAR, PXR, PPARs via carotenoid metabolites), which are poorly understood in relation to carotenoid health benefits and disease development in humans [216,217]. For instance, the activation of Nrf2, which regulates the cellular redox signaling and antioxidant defense, including carotenoids [162], can enhance the survival of cancer cells [218], which should be a topic of future investigations.

In a pilot study of 20 patients that were suffering from various cancers, it was shown that the concentration of carotenoids in the SC of patients that were measured before chemotherapy was significantly lower than that of healthy individuals (*p* < 0.001) [219], probably due to the psychological burden and anxiety that cancer patients suffered from or the accumulating long-lasting effect of previous chemotherapy cycles [220]. Although the concentration of carotenoids in the SC does not correlate with the age of a healthy person [192], such a correlation has been found for the skin of patients suffering from cancer, which was decreasing with increasing patient age [221].

Thus, it could be concluded that the action of internal stressors, including various diseases, leads to a decrease in the concentration of carotenoids in the SC, which entails a decrease of the entire antioxidant status.

### 5.6. Effect of Chemotherapy

Intravenously- and orally-applied chemotherapeutic agents against cancer diseases can reach the viable skin cells through the bloodstream and also penetrate into the SC through the secretion of sweat on the skin surface. Various substances are continuously eliminated from the body through the sweat; it was shown that pegylated liposomal doxorubicine, which is a cytostatic chemotherapeutic agent, and its metabolic products can be found on the skin surface after intravenous application in vivo by measuring a distinct fluorescence using confocal laser scanning microscopy [222]. Doxorubicine and its metabolites appeared within the sweat glands spreading continuously over the skin surface of palms and soles, which exhibit a high density of sweat glands. The fluorescent signal of doxorubicine could be detected 1–2 h after starting the intravenous infusion and remained there for at least 4.5 h [223] until it disappeared. Other non-fluorescent drugs, such as paclitaxel, 5-fluorouracil, and folinic acid, despite the presence of specific Raman bands [224], could not be detected on the skin surface in vivo by CRM, which may be due to their chemical properties, low concentration, and/or metabolism [219]. Before the invention of targeted therapies and immune therapy, many common chemotherapeutic agents were based on or caused radical formation, e.g., cytostatics inducing in particular ROS, increasing the amount of oxidation products in the body [225,226]. Cutaneous antioxidants neutralize part of the chemotherapy-induced free radicals and are further destroyed. Thus, the higher the concentration of antioxidants in the patient’s skin, the more effective the neutralization of free radicals and protective potential regarding skin toxicities at least locally, since the chemotherapy reaches the SC.

A chemotherapy including intravenous infusion of paclitaxel (175 mg/m^2^ every 3-weeks), docetaxel (30–35 mg/m^2^ every 3-weeks), or 5-fluorouracil (2400 mg/m^2^ every 2-weeks), leads to a decrease (*p* < 0.05) of the carotenoid concentration in the palm immediately after a single infusion by approximately 5, 12, and 7% on average, respectively, which is an indirect indicator of increased oxidative processes [221]. At the same time, the effect of non-fluorescent chemotherapeutic drugs on the palm was detected not only by a decrease in the carotenoid concentration, but also by an increase in the fluorescence intensity of the SC after the drug infusion [219], which can be explained by the oxidation of SC components caused by the action of the chemotherapeutic drug. This explanation is confirmed by recent studies [112,113], showing that the increase in the fluorescence intensity may be due to an increase in the concentration of oxidation products, such as proteins, lipids, amino acids, and DNA.

Thus, it has been shown, directly and indirectly, that systemically applied chemotherapeutic agents can reach the skin surface as part of the sweat, where they re-penetrate into the SC, interacting with its components, and reducing the carotenoid concentration [219,221].

### 5.7. Development of Hand-Foot Syndrome

The inability of the skin to effectively neutralize the formed ROS may be one of the probable causes of the chemotherapy-induced development of palmar-plantar erythrodysaesthesia (PPE), also known as hand-foot syndrome. PPE is a toxic side effect of various cancer treatments, manifesting as inflammatory skin eruptions of varying severity mainly on the skin of the palms and soles. Here, depending on the grade of severity, dryness, erythema, swelling or even blistering, and ulceration can be found, which can cause pain and considerable discomfort to the affected patients [227]. Hackbarth et al. [228] found that treatment with pegylated liposomal doxorubicine (40 mg/m^2^ every 4-weeks) causes manifestation of PPE in 84% of patients.

Considering the pathophysiology of PPE and the knowledge about the re-penetration of the chemotherapeutic agent into the SC through the sweat, a topical treatment option was developed involving the topical application of a cream with a high radical protection factor. It was assumed that the preventive topical application of antioxidants can neutralize the formed ROS within the SC before they can destroy cells or cell compartments. In a pilot study on 20 patients that were diagnosed with ovarian cancer that were treated with pegylated liposomal doxorubicine (40 mg/m^2^, 4 cycles) [229], it was shown that the topical application of a cream containing a balanced antioxidant mixture (Mapisal, Medac GmbH, Wedel, Germany) twice daily can significantly reduce the occurrence and severity of PPE [230]. The antioxidant mixture that was used in the cream was characterized by a very high free radical protection factor of 4500 × 10^14^ radicals/mg. For comparison, this value varies between (30–460) × 10^14^ radicals/mg for most sunscreens containing antioxidants [231]. Furthermore, a preventive effect of the antioxidant ointment against PPE was shown in another randomized, placebo-controlled double-blind study of 32 cancer patients, who were treated with pegylated liposomal doxorubicine. PPE mostly developed after two to four cycles of chemotherapy and not only the incidence but also the severity of PPE was lower after topical application of the antioxidant ointment compared to the placebo treatment [232].

The effect of reducing the incidence and severity of PPE appears to be related to the effective neutralization of free radicals that are produced on the skin surface by the release of the chemotherapeutic drug and/or its metabolites. These results are supported by studies showing that the topical administration of various antioxidants [233,234], as well as oral administration of vitamin E (300 mg/day) [235], significantly reduces the incidence and severity of PPE. Vitamin E can also reach the skin surface through the sweat secretion [53,54] and, acting as an antioxidant, neutralizes drug-induced free radicals.

The localization of skin defects in PPE can be explained by a high concentration of sweat glands [236] and the maximum thickness of the SC on the palms and soles [102]. Thus, the concentration of the chemotherapeutic agent that is released as part of the sweat will be the highest on the skin surface of the palms and soles. Furthermore, the drug penetrates into the SC, where it interacts with its components and accumulates in the thick SC, which serves as a reservoir for the penetrated substances. The thicker the SC, the greater the reservoir function is [237]. Accumulating in the SC, drug-induced free radicals destroy SC barrier structures, which may be one of the main reasons for rapid manifestation and development of PPE. A marked decrease in the manifestation of PPE that is caused by local ice cooling of the palms and soles [238] may be associated not only with a slowing down of the blood flow, but also with a decrease in sweating, which supports the previous hypothesis. Nevertheless, it is assumed that the decrease in sweating leads to a lower volume of the sweat secreted with a higher density of the chemotherapeutic agent (unpublished data). Another reason for the appearance of PPE on palmar-plantar skin sites might be the mechanical friction and pressure that might cause vascular microtrauma and enhance the inflammatory response and tissue damage combined with the local toxic effect of the chemotherapy [239].

It should be noted that the exact pathophysiologic mechanisms of PPE development remain unclear and are actively discussed by the scientific community. The development and severity of PPE will not only depend on the applied treatment agent, but also on individual physiological features, lifestyle habits, and circumstances. PPE development is a complex biochemical process that can involve all skin layers. Therefore, the antioxidant-based mechanism of PPE development in the SC [229,230] is not exclusive, but PPE development could also be initiated in the papillary dermis and the skin layers beneath, where the chemotherapeutic drug concentration is the highest due to the blood circulation. However, it should be considered that the pharmacokinetics and pharmacodynamics of chemotherapeutic drugs may not be the same for different skin layers and also strongly depend on the specific treatment agent. Thus, on the one hand, the time of drug release from the bloodstream and its accumulation by the cells of the viable epidermis and dermis is faster compared to the time of drug release to the skin surface with the sweat secretion (1–2 h, according to [222]). On the other hand, probably due to a higher concentration of various antioxidants (primarily enzymatic antioxidants), the cells of the viable epidermis and dermis might be more efficient in neutralizing the drug-induced free radicals, unlike the anucleate cells of the SC.

## 6. Factors Leading to an Increase of Carotenoids in the SC

The human body does not synthesize carotenoids [78,79] and their accumulation in the skin is entirely dependent on the uptake by nutrition containing these molecules. The diet must be balanced to increase the antioxidant activity and to eliminate the possible pro-oxidant effect that is observed when the physiological concentration of antioxidants is exceeded [27,48,86,240] or when the concentration of oxygen is increased [241]. Achieving a pro-oxidant effect is possible only through uncontrolled intake of dietary supplements containing high concentrations of individual antioxidants, which cannot be found in that amount and composition in natural plants. Therefore, the daily consumption of fruit and vegetables should be preferred to excessive intake of dietary supplements [27,216]. All fruit and vegetables contain antioxidants, including carotenoids and vitamins [70,242,243,244]. As demonstrated in paragraph 5, the carotenoid concentration in the SC decreases as a result of different stress factors. On the other hand, there is always a compensatory effect—the restoration of the carotenoid concentration in the SC, which is considerably slower compared to the degradation process [147,157,174,186] and strongly depends on nutrition and on the carotenoids that are stored in the body. For this reason, the most effective antioxidant protection of the human skin is reached by a combination of an antioxidant-rich nutrition and the topical application of antioxidants [48,245,246,247]. The effect of antioxidant-rich nutrition and topical formulations on the kinetics of carotenoids in the human SC are presented below being measured in vivo using one of the non-invasive methods that is presented in paragraph 3.

### 6.1. Effect of a Carotenoid-Rich Nutrition and Cosmetics on the Carotenoid Concentration in the SC

In vivo studies on humans show that the controlled consumption of dietary carotenoid-containing supplements leads to a significant dose-dependent increase in the carotenoid concentration in the blood plasma (on average by 200%) and in the SC (on average by 25%) [115,245,248,249]. The same effect is observed with the consumption of carotenoid-containing food, in particular fruit and vegetables [250,251,252,253,254,255]. At the same time, the carotenoid concentrations in the blood plasma and in the SC are strictly correlated [116,124,256,257,258]. Using RRS, it was shown in vivo that an 8-week course of daily oral intake of balanced natural plant antioxidants including vitamin E, vitamin C, polyphenols, carotenoids β-carotene, and lycopene, and other antioxidants, has a long-term effect of increasing the carotenoid concentration in the SC [245]. After finishing the intake, the carotenoid concentration in the SC is reduced to its initial value within 5-weeks, which exceeds the 2–4-weeks of the SC renewal. Therefore, it has to be assumed that the carotenoids saturate the SC by continuously diffusing from the storage in the body.

Using RRS, 10 healthy volunteers of Fitzpatrick skin Types II and III [259] were investigated in vivo under daily oral intake of a blue-green algae extract (*Spirulina platensis*, IGV Institut für Getreideverarbeitung GmbH, Nutetal, Germany) at an amount of 1.4 g/day containing 2.14 mg/g β-carotene, 1.91 mg/g zeaxanthin, and other carotenoids with a total carotenoid intake of 4.61 mg/g) for 8-weeks. A significant increase of the carotenoid concentration in the SC that was measured on the palm by approximately 22 % (*p* < 0.001) was shown. A tendency (*p* = 0.33) to increase the concentration of collagen Type I in the papillary dermis of the inner forearm was also observed using in vivo two-photon tomography [260].

In another placebo-controlled randomized study using diffuse reflectance spectroscopy [261] in 29 healthy women of Fitzpatrick skin Type II [259], the daily oral supplementation of green cabbage extract over 5- and 10-months (BioActive Food, Bad Segeberg, Germany), a carotenoid-rich dietary supplement (a daily carotenoid dose is 1650 µg: 1290 µg lutein, 210 µg β-carotene, 90 µg lycopene, and 60 µg zeaxanthin) shows a significant increase (*p* < 0.05) of the carotenoid concentration in the SC of the palm by approximately 10–22%. The daily dose of carotenoid intake was significantly lower (approximately four-fold) than in the previous study [260]. In addition, a significant increase in the concentration of collagen Type I (*p* < 0.01) after 5- and 10-months of green cabbage extract supplementation was shown on the cheek (increase by approximately 18 and 17%, respectively) and the inner forearm (increase by approximately 20 and 12%, respectively) [261]. Thus, increased concentrations of carotenoids in the SC lead to slower degradation of collagen Type I and, probably, promote the production of new collagen Type I by fibroblasts in the papillary dermis. The observed effect may be indirect and described by the increased antioxidant ability against ROS and, as a result, a reduced oxidative load on fibroblasts and the components of the extracellular matrix. Since a reduced concentration of collagen Type I is an indicator of skin aging [262], these results are promising in developing antiaging strategies. The correlation between the concentrations of carotenoids in the SC and collagen Type I in the dermis has been previously shown [149] however, the relationship to oral carotenoid supplementation was reported for the first time [261].

The fastest way to increase the concentration of antioxidants in the SC is the topical application of a cream containing antioxidants [57,245,263]. The topical application of antioxidant increases the skin protection against the effects of IR [149,154,164,167,178] and visible [149,154,162,164] radiation and can even be effective against itching and inflammation in the skin of atopic dermatitis patients [264]. It was shown that the daily application of a cream containing antioxidants, including 0.2% carotenoids, for 8-weeks has a short-term effect. After termination of the cream application the carotenoid concentration in the SC is reduced to the initial value within 10-days [245]. It has been demonstrated that combined oral and topical application of a carotenoid-enriched supplementation and ointment leads to the most efficient and prolonged (up to 5-weeks) increase in the concentration of carotenoids in the SC [245].

These results are in agreement with the current data in the literature [194,265]. In several in vivo studies [21,265,266,267], despite of the demonstrated protective effect, information about the effect of carotenoids on the antioxidant status of the SC was lacking.

### 6.2. Effect of a Carotenoid-Rich Nutrition on the Antioxidant Status of the SC

A double-blind, placebo-controlled, randomized clinical in vivo study involved 24 healthy volunteers of Fitzpatrick skin Types II or III [259], which were divided into two groups of 12 people, each. For 8-weeks, the first group took the dietary supplement Lutex skin^TM^ (BioActive Food GmbH, Bad Segeberg, Germany; the daily dose: 2200 µg lutein, 700 µg zeaxanthin, 1000 µg β-carotene, 50 µg α-carotene, 400 µg lycopene, and 100 µg cryptoxanthin, i.e., 4.45 mg of carotenoids in total), while the second group took an antioxidant-free placebo. The study lasted 20-weeks and included four control measurements: before the start of the dietary intervention at baseline, 4- and 8-weeks after the start of the intervention, and 12-weeks after the end of the intervention. In vivo measurements of the carotenoid concentration and the antioxidant status of the SC were performed on the inner forearm using RRS and EPR, respectively.

**Figure 6:**
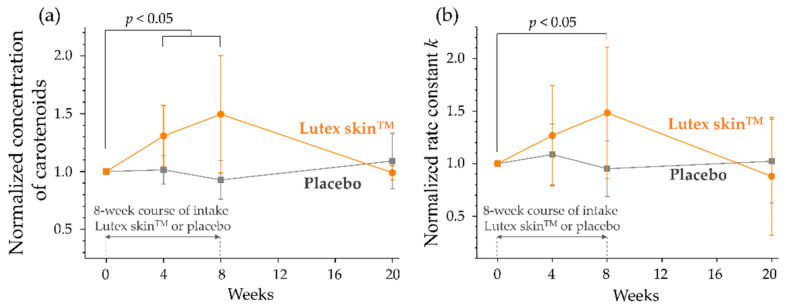
Carotenoid concentrations normalized to baseline (**a**) and the rate constant *k* determining the antioxidant status (**b**) that was measured in vivo in the SC of the inner side of the human forearm, of the verum group with the dietary supplementation Lutex skin^TM^ (*n* = 10, orange) and the placebo group (*n* = 12, grey) using RRS and EPR spectroscopy, respectively. The figure is adapted from [191].

The intake of the verum supplement within 4- and 8-weeks resulted in a significant increase in the carotenoid concentration in the SC (*p* < 0.05), which is in agreement with previous results with a 4-weeks supplementation (9 mg carotenoids/day) [248]. A total of 12-weeks after the termination of the dietary supplementation, the carotenoid concentration in the SC had decreased to the initial baseline level. In the placebo group the carotenoid concentrations in the SC did not significantly change over the total duration of the study (Figure 6a).

**Figure 7:**
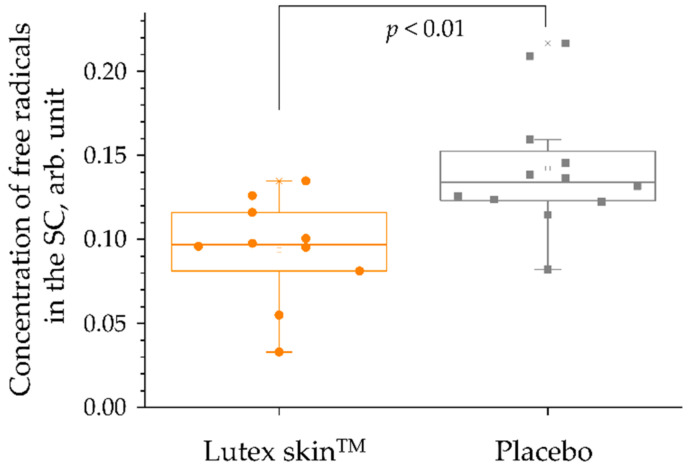
Concentration of free radicals that formed in the SC of the human inner forearm skin after irradiation with a sunlight simulator in the 420–2000 nm spectral range for the verum group under supplementation with Lutex skin^TM^ for 8-weeks (*n* = 10, orange color) and the placebo group (*n* = 12, grey color), measured in vivo by EPR spectroscopy. The figure is adapted from [191].

Figure 6b shows the effect of the verum supplementation on the rate constant *k*, which determines the antioxidant status of the human SC (Paragraph 4). It can be seen that the rate constant *k* correlates with the carotenoid concentration: unchanged in the placebo group, increasing after 4- and 8-weeks in the verum group, and decreasing to the initial value 12-weeks after the intake termination. After an 8-week intake of the verum supplement, when the difference in the carotenoid concentration and antioxidant status in the SC was the highest between the two groups, all the volunteers were exposed to a sunlight simulator in the broad 420–2000 nm spectral range (dose 72 J/cm^2^: light intensity 120 mW/cm^2^; exposure time 10 min) to induce free radicals. The free radicals were recorded in vivo by EPR spectroscopy using the stable nitroxide spin probe PCA (3-carboxy-2,2,5,5-tetramethyl-1-pyrrolidine-1-oxyl), which does not react with antioxidants of the human epidermis [43]. The results show that the amount of free radicals that were generated in the SC is significantly lower (by approximately 34%) in the verum group than in the placebo group (*p* < 0.01) (Figure 7). This effect is related to the antioxidant property of carotenoids to neutralize free radicals (primarily ROS), further confirming the increased antioxidant status of the SC in the verum compared to the placebo group. These results explain the reduction of the UV-B light-induced erythema intensity, which had been reported in other studies for the group of volunteers taking carotenoid supplements [120,186,268].

Thus, the results of this study further support the conclusion that carotenoids can be considered as marker substances for the entire antioxidant status of the human SC in vivo [131]. Consequently, a diet that is enriched with carotenoids leads to an increase in their concentration in the skin, and also to an increase in the antioxidant status of the SC [191].

### 6.3. Effect of Vitamin C- and Polyphenol-Enriched Nutrition on the Antioxidant Status of the SC

Vitamins, particularly ascorbic acid (vitamin C), have an antioxidant function in the human skin [269]. Vitamin C is found in fruits and vegetables and is one of the main antioxidants in the human diet [270]. It is also found in fruit juices and smoothies due to its preservative properties [244]. An in vivo study using EPR and RRS was performed in three groups of healthy volunteers: the placebo group with an oral intake of dextrose, which has no antioxidant properties, (group 1, *n* = 11); Group 2 with a daily supplementation of vitamin C at a concentration of 8.3 mg/g (Group 2, *n* = 11); and Group 3 with 14.9 mg/g vitamin C supplementation (Group 3, *n* = 11). After a 4-week course of supplementation, the rate constant *k* significantly increased (*p* < 0.05) up to 22% and 37% for the Groups 2 and 3, respectively, and remained unchanged in Group 1 (*p* > 0.05) [271], which signifies an increase in the antioxidant status of the SC in Groups 2 and 3. RRS investigations also showed an increase in carotenoid concentrations in the SC in both groups after vitamin C administration compared to the placebo group as a marked trend (*p* < 0.1).

The effects of an increased antioxidant status (*p* < 0.05) and a marked trend towards increased carotenoid concentrations (*p* > 0.1) in the human SC were observed over a 2-week course of a polyphenol-rich supplementation (mostly catechin and flavanols) of green tea [272] and a 4-week course of administration of a polyphenol-rich aronia peel extract that was stabilized with vitamin C [273]. The observed effect appears to be related to an increase in the concentration of polyphenols in the SC, which act synergistically with other antioxidants [274], and, as a result, an improvement in the protective function against ROS, as described in the literature [44,275]. The increased concentration of carotenoids in the SC after vitamin C or polyphenol administration is important for understanding the functional effects of the antioxidant system of the SC and once again proves that skin antioxidants are a well-balanced protective complex, having a synergistic effect, in which an increase or decrease in one antioxidant type can cause an adaptive response of other antioxidants, i.e., causing an increase or decrease in their concentration.

The photo-protective effect on human skin that is exerted by oral administration of various antioxidants has long been known, and has been measured by assessing the changes in the minimal erythema dose, i.e., minimally invasive [276,277,278]. The results show that a diet that is rich in antioxidants (particularly carotenoids) is important for their accumulation in the SC, which increases the antioxidant status of the SC and provides photo-protection for collagen Type I preventing the development of premature skin aging [149].

Currently, the terms “oral photo-protection” [279] or “nutritional protection” [280] are widely used, implying an increase in skin photo-protection through the consumption of antioxidant-containing products and/or dietary supplements for antiaging [279] and skin cancer prevention [281]. However, the increase of sun protection factor that is induced by endogenous carotenoids is low and skin photo-protection is mainly induced by the antioxidant properties of carotenoids [265], thus the topical application of sunscreens is recommended for optimal UV protection [282]. Nowadays, many sunscreens contain antioxidants enhancing the minimal erythema dose, thus increasing their sun protection factor.

### 6.4. Biofeedback as Motivation for a Healthier Lifestyle

Taking into account the importance of a daily diet of antioxidant-containing food, an in vivo study was carried out to investigate the use of cutaneous antioxidant measurements as an instrument of biofeedback in adolescents, where changes in the measurement value represent positive or negative lifestyle factors that can be influenced by the individual behavior. Here, diffuse reflectance spectroscopy was used to investigate changes in the concentration of carotenoids in the SC of the palm in 50 high school students that were aged from 17 to 20 years. Before starting the study, basement values of the cutaneous carotenoids were assessed. Afterwards, an intervention was conducted; the students received lectures on the importance of a healthy nutrition and lifestyle. Furthermore, the function of antioxidants and the factors influencing its change were explained, which subsequently served as a motivating factor for a healthier lifestyle. Measurements were taken at school at lunchtime twice a week for 7-months and after each measurement a questionnaire was completed including an assessment of the amount of fruit and vegetables in the daily diet, the current subjective stress level (including social and personal stress, exams, lack of sleep), health status (illness, fatigue), smoking, and alcohol consumption. The results showed that the concentration of carotenoids in the skin significantly increased after the intervention was conducted and even lasted beyond that during the following months with some subjects stating that they subjectively felt the positive effects on their health status being more energetic and having an improved skin appearance. It was seen that a rise in antioxidant levels correlated with a higher uptake of fruit and vegetables in the daily diet and with a reduction or avoidance of stress and smoking [283].

Another important result of this study was the observation of an even competitive motivation for higher antioxidant values among the participants. Comparing their skin carotenoid values with each other, the students started to compete for higher concentrations, thus striving for a healthier lifestyle. In order to achieve higher antioxidant values, most participants partially or completely abstained from smoking and drinking alcohol and increased the amount of fruit and vegetables in their daily diet. The rapid and non-invasive feedback by their antioxidant values gave the participants additional motivation to take part in the study and to improve their dietary and lifestyle habits [283]. The observations in this study are in good agreement with previous findings in adult volunteers who had participated in daily non-invasive measurements of cutaneous carotenoids for one year. Having a low concentration of carotenoids in the skin, some participants decided to change their lifestyle by reducing stress and improving the quality of their diet [52].

## 7. Conclusions

This review outlines the basics of redox regulation in human skin. A list of endogenous and exogenous skin antioxidants is presented and the possibility of their synergistic action is described. Particular attention is paid to carotenoids—antioxidants that can be measured non-invasively and in vivo in the human SC using optical methods, such as resonance Raman spectroscopy and diffuse reflectance spectroscopy.

The following main conclusions can be drawn:

1. Resonance Raman spectroscopy and diffuse reflectance spectroscopy are optical methods that are optimally suited for in vivo measurements of the carotenoid concentration in the human SC. They provide a rapid and non-invasive screening of the kinetics of carotenoids and changes in the antioxidant status of the human SC, which can be useful in skin research as well as clinical practice.
2. Carotenoids having ≥10 π-π-conjugated C=C double bonds serve as an objective marker of the antioxidant status of human SC in vivo according to the principle: the higher the concentration of carotenoids the higher the antioxidant status of the entire SC and vice versa.
3. In the SC, different exogenous and endogenous antioxidants form a well-balanced protective complex, which is characterized by a synergistic effect. An increase or decrease in one antioxidant substance can cause an adaptive response from other antioxidant substances, i.e., causing an increase or decrease in their concentration.
4. The exposure to doses of radiation in the visible (>50 J/cm^2^) and near-infrared (>120 J/cm^2^) spectral ranges cause the formation of free radicals (in particular, ROS) in human skin, which can be determined in vivo by a decrease in the concentration of carotenoids in the SC. The topical application of sunscreen containing antioxidants has a protective effect on the skin by neutralizing the formed free radicals.
5. A diet containing antioxidants, in particular fruit and vegetables or food supplements, leads to an increase in the carotenoid concentration and the antioxidant status of the human SC. The oral consumption of carotenoids increases photo-protection of human skin. Antioxidant uptake by natural sources, such as unprocessed fruit and vegetables is considered preferable to industrially processed antioxidants of dietary supplements.
6. The concentration of carotenoids in the SC can reflect the individual lifestyle habits and health status. For example, smokers, subjects consuming a low amount of fruit and vegetables, and patients under certain chemotherapeutic treatments have a significantly lower concentration of carotenoids in the SC than non-smoking healthy volunteers whose daily diet includes fruit and vegetables.
7. One of the probable pathophysiological mechanisms of the hand-foot syndrome, often associated with chemotherapy in cancer patients, is the reduced antioxidant status of the SC and the inability to neutralize the chemotherapeutics-induced free radicals. The topical application of antioxidants can neutralize free radicals that are formed on the skin surface by chemotherapeutic agents being released with the sweat. As a result, the frequency and severity of hand-foot syndrome are reduced.

## Abbreviations

CRM	confocal Raman micro-spectroscopy
IR	infrared (radiation, 760–3000 nm)
IR-A	infrared-A (radiation, 760–1440 nm)
IR-B	infrared-B (radiation, 1440–3000 nm)
MSRRS	multiple spatially resolved reflectance spectroscopy
NF-κB	Nuclear factor kappa-light-chain-enhancer of activated B cells
Nrf2	nuclear factor erythroid 2-related factor 2
PPARs	peroxisome proliferator-activated receptor
PXR	retinoid X receptor
RAR	retinoic acid receptor
ROS	reactive oxygen species
RRS	resonance Raman spectroscopy
SC	stratum corneum
TEMPO	2,2,6,6-tetramethylpiperidine-1-yl-oxyl
UV	ultraviolet (radiation, 290–400 nm)
UV-A	ultraviolet-A (radiation, 320–400 nm)
UV-B	ultraviolet-B (radiation, 290–320 nm).
